# Supplementary material for: Double‐Edged Role of Resource Competition in Gene Expression Noise and Control
Source: Adv Genet (Hoboken). 2022 Feb 8;3(1):2100050. doi: 10.1002/ggn2.202100050 (PMC9390979; doi:10.1002/ggn2.202100050)
Supplement: Supplementary file 1 — Supporting Information [file GGN2-3-2100050-s001.pdf]

Double-edged role of resource competition in gene expression noise and control

Hanah Goetz[1,+] *Austin Stone*[2,+] *Rong Zhang*[2] *Ying-Cheng Lai*[3,4] *Xiao-Jun Tian*[2,*]

[1]*School for Engineering of Matter, Transport and Energy, Arizona State University, Tempe, AZ, USA*

[2]*School of Biological and Health Systems Engineering, Arizona State University, Tempe, AZ, USA*

[3]*School of Electrical, Computer and Energy Engineering, Arizona State University, Tempe, AZ, USA*

[4]*Department of Physics, Arizona State University, Tempe, Arizona 85287, USA.*

[+]*These authors contributed equally to this work.*

[*]*Corresponding Author: xiaojun.tian@asu.edu.*

Supporting Information

A Models for systems without controllers

Two identically regulated genes are placed within the same circuit, as shown in Figure 1a. To differentiate between the two genes, fluorescent protein is used for the sole purpose of distinguishing protein expression. The first gene results are shown through GFP protein expression, while the second uses RFP protein. Three separate scenarios are considered for this gene circuit: unlimited resources (UR), resource competition (RC), and orthogonal resources (OR). The system is generally described by a set of ordinary differential equations:

$$\frac{dm_i}{dt} = J^+_{m_i} - J^-_{m_i}, \tag{1}$$

$$\frac{dp_i}{dt} = J^+_{p_i} - J^-_{p_i}, \tag{2}$$

where m_i and p_i represent the mRNA and the protein concentrations, respectively, with the subscripts $i = 1, 2$ indicating the first or the second mRNA/protein (GFP, RFP), $J^+_{m_i}$ and $J^-_{m_i}$ are respectively the production and degradation rates of mRNAs, and the respective rates for proteins are $J^+_{p_i}$ and $J^-_{p_i}$. The degradation rates of mRNAs and proteins are proportional to their concentration: $J^-_{m_i} = d_{m_i} m_i$ and $J^-_{p_i} = d_{p_i} p_i$, where d_{m_i} and d_{p_i} are constants of degradation. The forms of $J^+_{m_i}$ and $J^+_{p_i}$ depend on the assumptions used in each particular system.

A system with unlimited resources (UR) has absolutely no competition and can be modeled by

$$\frac{dm_i}{dt} = k_{m_i} R_i - d_{m_i} m_i, \tag{3}$$

$$\frac{dp_i}{dt} = k_{p_i} m_i - d_{p_i} p_i, \tag{4}$$

where k_{m_i} and k_{p_i} are the production rate constants, d_{m_i} and d_{p_i} are the degradation rate constants for the mRNAs and proteins, respectively, and R_i represents the effective plasmid DNA copy number. We thus have $J^+_{m_i} = k_{m_i} R_i$ and $J^+_{p_i} = k_{p_i} m_i$. Setting these derivatives to zero gives the steady-state mRNA

and protein mean concentrations, respectively, as

$$\langle m_i \rangle = \frac{k_{m_i} R_i}{d_{m_i}}, \tag{5}$$

$$\langle p_i \rangle = \frac{k_{p_i} k_{m_i} R_i}{d_{p_i} d_{m_i}}. \tag{6}$$

This type of model has been widely used to simulate gene expression levels and the corresponding noise [23, 24, 25, 26, 27, 28, 29, 30].

A system with limited shared resources causing resource competition (RC) stipulates the genes compete with both themselves and each other for the limited transcriptional and translation resources. The equations for this system [6] are

$$\frac{dm_i}{dt} = \frac{k_{m_i} R_i}{1 + \sum_j \frac{R_j}{J_{m_j}}} - d_{m_i} m_i \tag{7}$$

$$\frac{dp_i}{dt} = \frac{k_{p_i} m_i}{1 + \sum_j \frac{m_j}{J_{p_j}}} - d_{p_i} p_i \tag{8}$$

where J_{m_i} and J_{p_i} are the effective transcriptional and translational capacities of limited resources in the host cell for synthetic gene circuits, respectively. Mathematically, $J_m = \frac{k_m + k_r^{RNAP}}{k_f^{RNAP}}$ and $J_p = \frac{k_P + k_r^{Ribosome}}{k_f^{Ribosome}}$, as defined in [6], where k_m and k_p are the transcription and translation rate constants, k_r^{RNAP} and k_f^{RNAP} are the binding/unbinding rate constants of the RNAP to synthetic genes, and $k_r^{Ribosome}$ and $k_f^{Ribosome}$ are the binding/unbinding rate constants of the ribosome to mRNA respectively. Note that as J_{m_i} and J_{p_i} approaches infinity, the RC model reduces the UR model, so

$$J^+_{m_i} = \frac{k_{m_i} R_i}{1 + \sum_j \frac{R_j}{J_{m_j}}},$$

$$J^+_{p_i} = \frac{k_{p_i} m_i}{1 + \sum_j \frac{m_j}{J_{p_j}}}.$$

Again, setting these derivatives to zero yields the steady state mRNA and protein mean concentrations as

$$\langle m_i \rangle = \frac{k_{m_i} R_i}{d_{m_i} \left(1 + \sum_j \frac{R_j}{J_{m_j}}\right)}, \tag{9}$$

$$\langle p_i \rangle = \frac{k_{p_i} \langle m_i \rangle}{d_{p_i} \left(1 + \sum_j \frac{m_j}{J_{p_j}}\right)}. \tag{10}$$

A system with orthogonal resources (OR) eliminates the resource competition between genes, where the two genes still compete with themselves due to the limitation in the resources but not with each other. The system is described by

$$\frac{dm_i}{dt} = \frac{k_{m_i} R_i}{1 + \frac{R_i}{J_{m_i}}} - d_{m_i} m_i, \tag{11}$$

$$\frac{dp_i}{dt} = \frac{k_{p_i} m_i}{1 + \frac{m_i}{J_{p_i}}} - d_{p_i} p_i, \tag{12}$$

with $J^+_{m_i} = (k_{m_i} R_i)/(1 + R_i/J_{m_i})$ and $J^+_{p_i} = (k_{p_i} m_i)/(1 + m_i/J_{p_i})$. In the steady state, the mRNA and

protein mean concentrations are, respectively,

$$\langle m_i \rangle = \frac{k_{m_i} R_i}{d_{m_i} \left(1 + \frac{R_i}{J_{m_i}}\right)}, \tag{13}$$

$$\langle p_i \rangle = \frac{k_{p_i} \langle m_i \rangle}{d_{p_i} \left(1 + \frac{m_i}{J_{p_i}}\right)}. \tag{14}$$

The deterministic simulation shown in Figure 1c, 1e, 2b and Supplementary Figure 1c, 2a are based on this method.

B Gillespie stochastic simulations

To simulate the stochastic trajectories of mRNA and protein levels, we use the standard Gillespie stochastic simulation method [31], with the following steps.

1. Define the system according to expression levels using the following matrices: $\mathcal{S}$ - the number of each molecular used as reactants and $\mathcal{P}$ - the number of each molecular made as products :

$$\mathcal{S} = \begin{bmatrix} 0 & 0 & 0 & 0 \\ 1 & 0 & 0 & 0 \\ 1 & 0 & 0 & 0 \\ 0 & 1 & 0 & 0 \\ 0 & 0 & 0 & 0 \\ 0 & 0 & 1 & 0 \\ 0 & 0 & 1 & 0 \\ 0 & 0 & 0 & 1 \end{bmatrix}, \quad \mathcal{P} = \begin{bmatrix} 1 & 0 & 0 & 0 \\ 0 & 0 & 0 & 0 \\ 1 & 1 & 0 & 0 \\ 0 & 0 & 0 & 0 \\ 0 & 0 & 1 & 0 \\ 0 & 0 & 0 & 0 \\ 0 & 0 & 1 & 1 \\ 0 & 0 & 0 & 0 \end{bmatrix} \tag{15}$$

2. Initialize $\mathbf{n}$ as the vector of the mean copy numbers of mRNAs and proteins, $\mathbf{n} = [M_1, P_1, M_2, P_2]$ and set time $t = 0$. It is noted that we use capitalized letters M_i, P_i to denote the numbers of mRNAs and proteins, which can be converted to the concentrations $m_i = M_i/\Omega$ and $p_i = P_i/\Omega$ by using a system size factor Ω.

3. Update the reaction rate vector $\mathbf{v}$ using current $\mathbf{n}$:

$$\mathbf{v} = \left[J^+_{m_1}, J^-_{m_1}, J^+_{p1}, J^-_{p1}, J^+_{m2}, J^-_{m2}, J^+_{p2}, J^-_{p2}\right]^T. \tag{16}$$

4. Calculate the reaction probability vector:

$$\mathbf{a} = \frac{\mathbf{v}}{\sum_i v_i}, \tag{17}$$

where v_i is the ith component of $\mathbf{v}$.

5. Reaction r occurs if

$$P(r-1) < r_1 < P(r) \tag{18}$$

and the time step to the next reaction is

$$\tau = \frac{1}{a_0} \log \left(\frac{1}{r_2} \right) \tag{19}$$

where $a_0 = \sum_i v_i$, $P(r) = \sum_{i=1}^{r} a_i$, a_i is the ith component of $\mathbf{a}$,

r_1 and r_2 are two uniformly distributed random numbers from the unit interval $[0, 1]$.

6. Update $\mathbf{n}$ and t according to

$$\begin{aligned} n_i &\to n_i - \mathcal{S}_{r,i} + \mathcal{P}_{r,i}, \\ t &= t + \tau \end{aligned} \tag{20}$$

7. Continue steps 3-6 until t reaches a predetermined maximum time.

The stochastic simulation shown in Figure 1c, 1e, 2b and Supplementary Figure 1b, 2a, 9a-k is based on this method.

C Master equation

The probability $P(M_1, P_1, M_2, P_2, t)$ of the system in the state (M_1, P_1, M_2, P_2, t) at time t is governed by the master equation:

$$\begin{aligned} &\frac{dP\left(M_1, P_1, M_2, P_2\right)}{dt} \\ &= J_{m_1}^{+}\left(M_1 - 1, P_1, M_2, P_2\right) P\left(M_1 - 1, P_1, M_2, P_2\right) + J_{m_1}^{-}\left(M_1 + 1, P_1, M_2, P_2\right) P\left(M_1 + 1, P_1, M_2, P_2\right) \\ &+ J_{P_1}^{+}\left(M_1, P_1 - 1, M_2, P_2\right) P\left(M_1, P_1 - 1, M_2, P_2\right) + J_{P_1}^{-}\left(M_1, P_1 + 1, M_2, P_2\right) P\left(M_1, P_1 + 1, M_2, P_2\right) \\ &+ J_{m_2}^{+}\left(M_1, P_1, M_2 - 1, P_2\right) P\left(M_1, P_1, M_2 - 1, P_2\right) + J_{m_2}^{-}\left(M_1, P_1, M_2 + 1, P_2\right) P\left(M_1, P_1, M_2 + 1, P_2\right) \\ &+ J_{P_2}^{+}\left(M_1, P_1, M_2, P_2 - 1\right) P\left(M_1, P_1, M_2, P_2 - 1\right) + J_{P_2}\left(M_1, P_1, M_2, P_2 + 1\right) P\left(M_1, P_1, M_2, P_2 + 1\right) \\ &- \left[J_{m_1}^{+}\left(M_1, P_1, M_2, P_2\right) + J_{m_1}^{-}\left(M_1, P_1, M_2, P_2\right) + J_{P_1}^{+}\left(M_1, P_1, M_2, P_2\right) + J_{m_2}^{-}\left(M_1, P_1, M_2, P_2\right)\right. \\ &+ J_{m_2}^{+}\left(M_1, P_1, M_2, P_2\right) + J_{m_2}^{-}\left(M_1, P_1, M_2, P_2\right) + J_{P_2}^{+}\left(M_1, P_1, M_2, P_2\right) \\ &\left.+J_{P_2}^{-}\left(M_1, P_1, M_2, P_2\right)\right] P\left(M_1, P_1, M_2, P_2\right). \end{aligned} \tag{21}$$

Equation (21) can be written in matrix form as

$$\frac{d\mathbf{P}}{dt} = \mathcal{A} \cdot \mathbf{P}, \tag{22}$$

where $\mathbf{P}$ is the state probability vector, and $\mathcal{A}$ is the transition rate matrix from state $(M_1 + i, P_1 + j, M_2 + k, P_2 + l)$ to state (M_1, P_1, M_2, P_2), which is defined as

$$A\left(M_1 + i, P_1 + j, M_2 + k, P_2 + l \to M_1, P_1, M_2, P_2\right) =$$

$$\begin{cases} -\begin{bmatrix} J^+_{m_1}(M_1,P_1,M_2,P_2)+J^-_{m_1}(M_1,P_1,M_2,P_2) \\ +J^+_{P_1}(M_1,P_1,M_2,P_2)+J^-_{m_2}(M_1,P_1,M_2,P_2) \\ +J^+_{m_2}(M_1,P_1,M_2,P_2)+J^-_{m_2}(M_1,P_1,M_2,P_2) \\ +J^+_{P_2}(M_1,P_1,M_2,P_2)+J^-_{P_2}(M_1,P_1,M_2,P_2) \end{bmatrix} & (i=0,j=0,k=0,l=0) \\ J^+_{m_1}(M_1-1,P_1,M_2,P_2) & (i=-1,j=0,k=0,l=0) \\ J^-_{m_1}(M_1+1,P_1,M_2,P_2) & (i=1,j=0,k=0,l=0) \\ J^+_{p_1}(M_1,P_1-1,M_2,P_2) & (i=0,j=-1,k=0,l=0) \\ J^-_{p_1}(M_1,P_1+1,M_2,P_2) & (i=0,j=1,k=0,l=0) \\ J^+_{m_2}(M_1,P_1,M_2-1,P_2) & (i=0,j=0,k=-1,l=0) \\ J^-_{m_2}(M_1,P_1,M_2+1,P_2) & (i=0,j=0,k=1,l=0) \\ J^+_{p_2}(M_1,P_1,M_2,P_2-1) & (i=0,j=0,k=0,l=-1) \\ J^-_{p_2}(M_1,P_1,M_2,P_2+1) & (i=0,j=0,k=0,l=1) \\ 0 & \text{otherwise} \end{cases}$$

We solved the master equation until the system reached a steady-state distribution. The boundary was set to $[0, 3{*}\mu_i]$ so the probabilities at right and top boundaries would be small. $\mu_i = \{\langle M_1\rangle, \langle M_2\rangle, \langle P_1\rangle, \langle P_2\rangle\}$ is the mean number of mRNAs or proteins. The no-flux boundary conditions are used to conserve probability. The master equation is used in Figure 1d, 1g, 2c, and 2b.

The 2D and 1D protein distribution shown in Figure 1d, 1f, 1g, 2c-d and Supplementary Figure 2b-c are based on this method.

D Analytical solution from the normalized fluctuation-dissipation theorem

We define the noise (η) for both mRNAs and proteins as the coefficient of variation:

$$\eta_i = \sqrt{\frac{\sigma_{ii}}{{\mu_i}^2}} = \frac{\sigma_i}{\mu_i} \tag{23}$$

where $\sigma_i = \{\sigma_{m_1}, \sigma_{p_1}, \sigma_{m_2}, \sigma_{p_2}\}$ is the standard deviation (σ_{ii} as variance).

To obtain the analytical expressions of gene expression noise, we use the normalized version of the FDT [33]:

$$\mathcal{M} \cdot \mathcal{C}^* + \mathcal{C}^* \mathcal{M}^T + \mathcal{D} = 0, \tag{24}$$

where $C^*_{ij} = \sigma_{ij}/(\langle\mu_i\rangle\langle\mu_j\rangle)$ is the normalized covariance, $M_{ij} = -H_{ij}/\tau_i$ is the elements of the dynamical matrix with

$$H_{ij} = \frac{\partial \ln\left(\frac{J_i^-}{J_i^+}\right)}{\partial \ln n_j},$$

and $D_{ii} = 2/(n_i \tau_i)$. Here, n_i with $i = \{1, 2, 3, 4\}$ represents the copy number of m_1, p_1, m_2 and p_2, respectively.

For the resource competition (RC) system, the matrices $\mathcal{M}$ and $\mathcal{D}$ are defined as

$$\mathcal{M} = \begin{bmatrix} \frac{H_{11}}{\tau_1} & 0 & 0 & 0 \\ \frac{H_{21}}{\tau_2} & \frac{H_{22}}{\tau_2} & \frac{H_{23}}{\tau_2} & 0 \\ 0 & 0 & \frac{H_{33}}{\tau_3} & 0 \\ \frac{H_{41}}{\tau_4} & 0 & \frac{H_{43}}{\tau_4} & \frac{H_{44}}{\tau_4} \end{bmatrix}, \tag{25}$$

$$\mathcal{D} = \begin{bmatrix} \frac{2}{M_1\tau_1} & 0 & 0 & 0 \\ 0 & \frac{2}{P_1\tau_2} & 0 & 0 \\ 0 & 0 & \frac{2}{M_2\tau_3} & 0 \\ 0 & 0 & 0 & \frac{2}{P_2\tau_4} \end{bmatrix}. \tag{26}$$

The normalized FDT equation can be solved to yield the following noise levels of mRNAs and proteins:

$$\begin{aligned}
\eta^2_{m_1\text{total}} &= \frac{\sigma^2_{m_1}}{{M_1}^2} = \frac{1}{H_{11}M_1} \\
\eta^2_{p_1\text{ total}} &= \frac{\sigma^2_{p_1}}{{P_1}^2} = \frac{1}{H_{22}P_1} + \frac{\sigma^2_{m_1}}{{M_1}^2} \times \frac{{H_{21}}^2}{{H_{22}}^2} \times \frac{H_{22}/\tau_2}{(H_{11}/\tau_1 + H_{22}/\tau_2)} + \frac{\sigma^2_{m_2}}{{M_2}^2} \times \frac{{H_{23}}^2}{{H_{22}}^2} \times \frac{H_{22}/\tau_2}{(H_{33}/\tau_3 + H_{22}/\tau_2)} \\
\eta^2_{m_2\text{ total}} &= \frac{\sigma^2_{m_2}}{{M_2}^2} = \frac{1}{H_{33}M_2} \\
\eta^2_{p_2\text{ total}} &= \frac{\sigma^2_{p_2}}{{P_2}^2} = \frac{1}{H_{44}P_2} + \frac{\sigma^2_{m_2}}{{M_2}^2} \times \frac{{H_{43}}^2}{{H_{44}}^2} \times \frac{H_{44}/\tau_4}{(H_{33}/\tau_3 + H_{44}/\tau_4)} + \frac{\sigma^2_{m_1}}{{M_1}^2} \times \frac{{H_{41}}^2}{{H_{44}}^2} \times \frac{H_{44}/\tau_4}{(H_{11}/\tau_1 + H_{44}/\tau_4)}
\end{aligned} \tag{27}$$

with

$$\begin{aligned}
\mathrm{H}_{11} &= \frac{\partial \ln\left(J^-_{m_1}/J^+_{m_1}\right)}{\partial \ln(M_1)}, \quad \mathrm{H}_{22} = \frac{\partial \ln\left(J^-_{p_1}/J^+_{p_1}\right)}{\partial \ln(P_1)}, \quad \mathrm{H}_{33} = \frac{\partial \ln\left(J^-_{m_2}/J^+_{m_2}\right)}{\partial \ln(M_2)}, \quad \mathrm{H}_{44} = \frac{\partial \ln\left(J^-_{p_2}/J^+_{p_2}\right)}{\partial \ln(P_2)} \\
\mathrm{H}_{21} &= \frac{\partial \ln\left(J^-_{p_1}/J^+_{p_1}\right)}{\partial \ln(M_1)}, \quad \mathrm{H}_{23} = \frac{\partial \ln\left(J^-_{p_1}/J^+_{p_1}\right)}{\partial \ln(M_2)}, \quad \mathrm{H}_{41} = \frac{\partial \ln\left(J^-_{p_2}/J^+_{p_2}\right)}{\partial \ln(M_1)}, \quad \mathrm{H}_{43} = \frac{\partial \ln\left(J^-_{p_2}/J^+_{p_2}\right)}{\partial \ln(M_2)}.
\end{aligned}$$

The revised production and degradation rates taking into account the system size factor Ω are

$$J^+_{m_i} = \frac{k_{m_i}R_i}{1+\sum_j \frac{R_j}{J_{m_j}}}\Omega, \quad J^-_{m_i} = M_i d_{m_i}, \quad J^+_{p_i} = k_{p_i}\frac{M_i}{1+\sum_j \frac{M_j/\Omega}{J_{p_j}}}\Omega, \quad J^-_{p_i} = P_i d_{p_i}. \tag{28}$$

Substituting the parameter values of our model $H_{11} = 1$, $H_{22} = 1$, $H_{33} = 1$ and $H_{44} = 1$ into Eq. (27), we get

$$\begin{aligned}
\eta^2_{m_1\text{ total}} &= \frac{\sigma^2_{m_1}}{{M_1}^2} = \frac{1}{M_1}, \\
\eta^2_{p_1\text{ total}} &= \frac{\sigma^2_{p_1}}{{P_1}^2} = \frac{1}{P_1} + \frac{\sigma^2_{m_1}}{{M_1}^2} \times {H_{21}}^2 \times \frac{\frac{1}{\tau_2}}{\left(\frac{1}{\tau_1}+\frac{1}{\tau_2}\right)} + \frac{\sigma^2_{m_2}}{{M_2}^2} \times {H_{23}}^2 \times \frac{\frac{1}{\tau_2}}{\left(\frac{1}{\tau_3}+\frac{1}{\tau_2}\right)}, \\
\eta^2_{m_2\text{ total}} &= \frac{\sigma^2_{m_2}}{{M_2}^2} = \frac{1}{M_2}, \\
\eta^2_{p_2\text{ total}} &= \frac{\sigma^2_{p_2}}{{P_2}^2} = \frac{1}{P_2} + \frac{\sigma^2_{m_2}}{{M_2}^2} \times {H_{43}}^2 \times \frac{\frac{1}{\tau_4}}{\left(\frac{1}{\tau_3}+\frac{1}{\tau_4}\right)} + \frac{\sigma^2_{m_1}}{{M_1}^2} \times {H_{41}}^2 \times \frac{\frac{1}{\tau_4}}{\left(\frac{1}{\tau_1}+\frac{1}{\tau_4}\right)}
\end{aligned} \tag{29}$$

Thus after decomposition, the total noise of GFP has three parts: the stochasticity from the random

birth/death of protein (η_p), the fluctuations of the gene's own mRNA (η_m), and the fluctuations of the other mRNA (η_{RC}),

$$\eta_p^2 = \frac{\sigma_{p_1}^2}{{P_1}^2} = \frac{1}{P_1} \tag{30}$$

$$\eta_m^2 = \frac{\sigma_{m_1}^2}{{M_1}^2} \times {H_{21}}^2 \times \frac{\frac{1}{\tau_2}}{\left(\frac{1}{\tau_1} + \frac{1}{\tau_2}\right)} \tag{31}$$

$$\eta_{RC}^2 = \frac{\sigma_{m_2}^2}{{M_2}^2} \times {H_{23}}^2 \times \frac{\frac{1}{\tau_2}}{\left(\frac{1}{\tau_3} + \frac{1}{\tau_2}\right)} \tag{32}$$

It is noted that the η_m depends on J_p through H_{21} and η_{RC} depends on J_p through H_{23}. Analytically,

$$H_{21} = -\frac{J_{p_1}(m_2 + J_{p_2}\Omega)}{J_{p_1}m_2 + J_{p2}m_1 + J_{p_1}J_{p_2}\Omega} \tag{33}$$

$$H_{22} = \frac{J_{p_1}m_2}{J_{p_1}m_2 + J_{p_2}m_1 + J_{p_1}J_{p_2}\Omega} \tag{34}$$

For two identical genes, $J_{p_1} = J_{p_2} = J_p$, thus

$$H_{21} = -\frac{m_2 + J_p\Omega}{m_1 + m_2 + J_p\Omega}. \tag{35}$$

$$H_{23} = \frac{m_2}{m_1 + m_2 + J_p\Omega} \tag{36}$$

Thus η_m monotonically increases while η_{RC} decreases with J_p, as shown in Figure 1h.

For the unlimited resources (UR) and the orthogonal resources (OR) systems, the matrices $\mathcal{M}$ and $\mathcal{D}$ are given by

$$\mathcal{M} = \begin{bmatrix} \frac{H_{11}}{\tau_1} & 0 & 0 & 0 \\ \frac{H_{21}}{\tau_2} & \frac{H_{22}}{\tau_2} & 0 & 0 \\ 0 & 0 & \frac{H_{33}}{\tau_3} & 0 \\ 0 & 0 & \frac{H_{43}}{\tau_4} & \frac{H_{44}}{\tau_4} \end{bmatrix}, \quad \mathcal{D} = \begin{bmatrix} \frac{2}{m_1\tau_1} & 0 & 0 & 0 \\ 0 & \frac{2}{p_1\tau_2} & 0 & 0 \\ 0 & 0 & \frac{2}{m_2\tau_3} & 0 \\ 0 & 0 & 0 & \frac{2}{p_2\tau_4} \end{bmatrix}. \tag{37}$$

We then solve the normalized FDT equations to obtain

$$\begin{aligned} \eta_{m_1\text{ total}}^2 &= \frac{\sigma_{m_1}^2}{{P_1}^2} = \frac{1}{H_{11}P_1} \\ \eta_{p_1\text{ total}}^2 &= \frac{\sigma_{p_1}^2}{{P_1}^2} = \frac{1}{H_{22}P_1} + \frac{\sigma_{m_1}^2}{{M_1}^2} \times \frac{{H_{21}}^2}{{H_{22}}^2} \times \frac{H_{22}/\tau_2}{(H_{11}/\tau_1 + H_{22}/\tau_2)} \\ \eta_{m_2\text{ total}}^2 &= \frac{\sigma_{m_2}^2}{{M_2}^2} = \frac{1}{H_{33}M_2} \\ \eta_{p_2\text{ total}}^2 &= \frac{\sigma_{p_2}^2}{{P_2}^2} = \frac{1}{H_{44}P_2} + \frac{\sigma_{m_2}^2}{{M_2}^2} \times \frac{{H_{43}}^2}{{H_{44}}^2} \times \frac{H_{44}/\tau_4}{(H_{33}/\tau_3 + H_{44}/\tau_4)} \end{aligned} \tag{38}$$

with

$$\mathrm{H}_{11} = \frac{\partial \ln\left(J^-_{m_1}/J^+_{m_1}\right)}{\partial \ln\left(M_1\right)}, \quad \mathrm{H}_{22} = \frac{\partial \ln\left(J^-_{p_1}/J^+_{p_1}\right)}{\partial \ln\left(P_1\right)}, \quad \mathrm{H}_{33} = \frac{\partial \ln\left(J^-_{m_2}/J^+_{m_2}\right)}{\partial \ln\left(M_2\right)},$$
$$\mathrm{H}_{44} = \frac{\partial \ln\left(J^-_{p_2}/J^+_{p_2}\right)}{\partial \ln\left(P_2\right)} \quad \mathrm{H}_{21} = \frac{\partial \ln\left(J^-_{1}/J^+_{p_1}\right)}{\partial \ln\left(M_1\right)}, \quad \mathrm{H}_{43} = \frac{\partial \ln\left(J^-_{p_2}/J^+_{p_2}\right)}{\partial \ln\left(M_2\right)}.$$

For the unlimited resource (UR) system, we have

$$\begin{aligned} J^+_{m_i} &= k_{m_i} R_i \Omega, \quad J^-_{m_i} = M_i d_{m_i} \\ J^+_{p_i} &= k_{p_i} M_i \Omega, \quad J^-_{p_i} = P_i d_{p_i} \end{aligned} \tag{39}$$

For the orthogonal resource (OR) system, we have

$$\begin{aligned} J^+_{m_i} &= \frac{k_{m_i} R_i}{1 + R_i/J_{m_i}} \Omega, \quad J^-_{m_i} = M_i d_{m_i} \\ J^+_{p_i} &= k_{p_i} \frac{M_i}{1 + M_i/\Omega/J_{p_i}} \Omega, \quad J^-_{p_i} = P_i d_{p_i} \end{aligned} \tag{40}$$

Substituting $H_{11} = 1$, H_{22}=1, $H_{33} = 1$, and $H_{44} = 1$ into Eq. (38), we obtain

$$\begin{aligned} \eta^2_{m_1\ \mathrm{total}} &= \frac{\sigma^2_{m_1}}{{M_1}^2} = \frac{1}{M_1} \\ \eta^2_{p_1\ \mathrm{total}} &= \frac{\sigma^2_{p_1}}{{P_1}^2} = \frac{1}{P_1} + \frac{\sigma^2_{m_1}}{{M_1}^2} \times {H_{21}}^2 \times \frac{\frac{1}{\tau_2}}{\left(\frac{1}{\tau_1} + \frac{1}{\tau_2}\right)} \\ \eta^2_{m_2\ \mathrm{total}} &= \frac{\sigma^2_{m_2}}{{M_2}^2} = \frac{1}{M_2} \\ \eta^2_{p_2\ \mathrm{total}} &= \frac{\sigma^2_{p_2}}{{P_2}^2} = \frac{1}{P_2} + \frac{\sigma^2_{m_2}}{{M_2}^2} \times {H_{43}}^2 \times \frac{\frac{1}{\tau_4}}{\left(\frac{1}{\tau_3} + \frac{1}{\tau_4}\right)} \end{aligned} \tag{41}$$

It is noted that in UR system, $H_{21} = -1$ thus η_m does not change with J_p, as shown in Supplementary Figure 2d.

The simulation shown in Figure 1h, 2e-f and Supplementary Figure 1a, 2d, 3a-b are based on this method.

E Total noise in the RC case is always smaller than UR case.

Here we compare the total noise of GFP in the RC system and in UR system. We scaled the parameters so that we have the same mean numbers of proteins and mRNA in both cases. The first noise term is the same as it only depends on the protein number. The mRNA noise in the 2nd and/or 3rd term, which depends on the mean number of two mRNAs, are also the same. In addition, the time scale parameters in the 2nd and/or 3rd terms are the same for two identical genes. Thus we only need to compare the difference between the values of $H^2_{21} + H^2_{23}$ in RC case and H^2_{21} in the UR case. In the UR case, $H^2_{21} = 1$.

In the RC case, we have

$$\begin{aligned} H_{21}^2 + H_{23}^2 &= (\frac{m_2 + J_p\Omega}{m_1 + m_2 + J_p\Omega})^2 + (\frac{m_2}{m_1 + m_2 + J_p\Omega})^2 \\ &= (\frac{m_2 + J_p\Omega}{m_1 + m_2 + J_p\Omega})^2 + (\frac{m_1}{m_1 + m_2 + J_p\Omega})^2 \\ &< (\frac{m_1 + m_2 + J_p\Omega}{m_1 + m_2 + J_p\Omega})^2 \\ &= 1 = H_{21}^2. \end{aligned}$$

Thus, the total noise in the RC case is always smaller than UR case, as shown in Supplementary Figure 2d.

F Generalized model with negative feedback controllers

We considered three types of negative feedback controllers to mitigate the resource competition effects: local, global, and negatively competitive regulator (NCR) controllers. For each type of controller, depending on the mediators and targets of the negative feedback, there are four subtypes: mRNA inhibiting transcription (MIX), protein inhibiting transcription (PIX), mRNA inhibiting translation (MIL), and protein inhibiting translation (PIL). To model the noise resulting from embedding a negative feedback controller into the two-gene circuit, we modify the previous model [20] by including the translational step in protein biosynthesis. The system with both resource competition and control is described by

$$\frac{dm_i}{dt} = \frac{k_{m_i} R_i}{1 + \sum_j \frac{R_j}{J_{m_j}}} \times \frac{1}{C_{m_i}} - d_{m_i} m_i, \tag{42}$$

$$\frac{dp_i}{dt} = \frac{k_{p_i} m_i}{1 + \sum_j \frac{m_j}{J_{p_j}}} \times \frac{1}{C_{p_i}} - d_{p_i} p_i, \tag{43}$$

where C_{m_i} and C_{p_i} characterize the repression of transcription and translation by the controller, respectively, which are given by

$$C_{m_i} = \begin{cases} 1 + S_c[G_i + \lambda_{GC} \sum_j G_i(1-\delta_{ij})], & \text{MIX or PIX,} \\ 1, & \text{MIL or PIL,} \end{cases} \tag{44}$$

$$C_{p_i} = \begin{cases} 1 + S_c[G_i + \lambda_{GC} \sum_j G_i(1-\delta_{ij})], & \text{MIL or PIL,} \\ 1, & \text{MIX or PIX,} \end{cases} \tag{45}$$

where S_c is the strength of the controller, G_i represents each gene's contribution to the controller's negative feedback activity, which is given by

$$G_i \equiv \begin{cases} G_{m_i}, & \text{MIX or MIL} \\ G_{p_i}, & \text{PIX or PIL} \end{cases} \tag{46}$$

with G_{m_i} and G_{p_i} defined as

$$G_{m_i} \equiv \frac{m_i/J_i}{1 + \frac{m_i}{J_i} + (1-\lambda_{LC}) \sum_j \frac{m_j}{J_j}(1-\delta_{ij})} \tag{47}$$

$$G_{p_i} \equiv \frac{p_i/J_i}{1 + \frac{p_i}{J_i} + (1-\lambda_{LC}) \sum_j \frac{p_j}{J_j}(1-\delta_{ij})}. \tag{48}$$

In Eqs. (47) and (48), δ_{ij} is the Kronecker delta matrix, where $\delta = 1$ for $i = j$ and zero otherwise, and the binding affinity of the repressive complex is represented by J_i. The hyperparameters λ_{LC} and λ_{GC} in Eqs. (44), (45), (47), and (48) are given by

$$\lambda_{LC} = \begin{cases} 1, & \text{local or NCR Controller,} \\ 0, & \text{global Controller,} \end{cases}$$

$$\lambda_{GC} = \begin{cases} 1, & \text{global or NCR Controller,} \\ 0, & \text{local Controller.} \end{cases}$$

Similarly, the systems with orthogonal resource and controllers are described by

$$\frac{dm_i}{dt} = \frac{k_{m_i} R_i}{1 + \frac{R_i}{J_{m_i}}} \times \frac{1}{C_{m_i}} - d_{m_i} m_i, \quad (49)$$

$$\frac{dp_i}{dt} = \frac{k_{p_i} m_i}{1 + \frac{m_i}{J_{p_i}}} \times \frac{1}{C_{p_i}} - d_{p_i} p_i, \quad (50)$$

where C_{m_i} and C_{p_i} are given by Eqs. (44) and (45), respectively. Equations (42-50) model all twenty-four negative feedback and mixed controller types studied.

The deterministic simulation shown in Figure 4e-f and Supplementary Figure 9a-k are based on this method.

G Determining gene expression noise levels using fluctuation-dissipation theorem

To calculate the mRNA and protein noise levels with negative controllers, we used the fluctuation-dissipation theorem [32, 33]:

$$\mathcal{J} \cdot \mathcal{C} + \mathcal{C} \cdot \mathcal{J}^T + \mathcal{D} = 0, \quad (51)$$

where $\mathcal{C}$ is the correlation matrix with the covariance σ_{ij} and $(\sigma_{ii} = \sigma_i^2)$, $\mathcal{J}$ is the Jacobian matrix of all partial derivatives of the vector field, and $\mathcal{D}$ is the diffusion matrix. With $\mathbf{n}$ as the vector of molecule copy numbers and $\mathcal{N}$ as the stoichiometric matrix given by

$$\mathcal{N} = \begin{bmatrix} 1 & -1 & 0 & 0 & 0 & 0 & 0 & 0 \\ 0 & 0 & 1 & -1 & 0 & 0 & 0 & 0 \\ 0 & 0 & 0 & 0 & 1 & -1 & 0 & 0 \\ 0 & 0 & 0 & 0 & 0 & 0 & 1 & -1 \end{bmatrix}, \quad (52)$$

and the reaction rate vector $\mathbf{v}$,

the diffusion and Jacobian matrices can be obtained from

$$\mathcal{D} = \mathcal{N} \cdot \text{diag}(\mathbf{v}) \cdot \mathcal{N}^T,$$

$$\mathcal{J} = \mathcal{N} \cdot \frac{\partial \mathbf{v}}{\partial \mathbf{n}}.$$

To solve for the correlation matrix $\mathcal{C}$, we used an algorithm based on the Sylvester equation $(\mathcal{I} \otimes \mathcal{J} + \mathcal{J}^T \otimes \mathcal{I})\,\text{vec}(\mathcal{C}) = \text{vec}(-\mathcal{D})$, which gives

$$\text{vec}(\mathcal{C}) = \frac{\text{adj}(\mathcal{I} \otimes \mathcal{J} + \mathcal{J} \otimes \mathcal{I})}{\det(\mathcal{I} \otimes \mathcal{J} + \mathcal{J} \otimes \mathcal{I})}\,\text{vec}(-\mathcal{D}), \quad (53)$$

where adj denotes the adjugate matrix, det is the determinant, and $\mathcal{I}$ is the identity matrix. The Kronecker product $\otimes$ is used to find the matrix direct product. That is, for $\mathcal{A}$ an $m \times n$ matrix and $\mathcal{B}$ a $p \times q$ matrix, their Kronecker product $\mathcal{E} = \mathcal{A} \otimes \mathcal{B}$ is a $mp \times nq$ matrix that has elements defined by $e_{\alpha\beta} = a_{ij} b_{kl}$, where $\alpha = p_{i-1} + k$ and $\beta = q_{j-1} + 1$. After solving the Sylvester equation, $\mathrm{vec}(\mathcal{C})$ can be reshaped to the same dimensions as the Jacobian matrix, leading to the correlation matrix $\mathcal{C}$, which in turn gives the variance σ_{ii} and covariance σ_{ij}.

The simulation shown in Figure 3c-h, 4c-d, 5a-g and Supplementary Figure 4-8, 9h are based on this method.

H Average noise reduction coefficient

To assess the contribution of each controller modality to reduction of system noise, we define the quantity "average noise reduction coefficient," denoted as $\langle C_{n\downarrow} \rangle$. The controller dimensions taken into account when caluclating $\langle C_{n\downarrow} \rangle$ include the RFP mean expression, controller strength, controller type (local, global, NCR), controller subtype (MIX, PIX, MIL, PIL), and controller application system (RC or OR). Given a function $\phi(P_2, S_c, i, j, k)$ that returns noise for a given RFP mean (P_2), controller strength (S_c), and discrete dimensions of controller type (i), controller subtype (j), and controller application case (k), we define $\langle C_{n\downarrow} \rangle$ of a certain controller modality as the average reduction in noise summed across all dimensions, holding the modality constant. Depending on whether GFP noise or total circuit noise is being considered, ϕ can return either GFP noise or the Pythagorean sum of GFP and RFP noise. We perform this operation after normalizing ϕ to the base case through $\phi_{\text{normalized}} = \phi_{\text{experimental case}} / \phi_{\text{base case}}$. These considerations lead to

$$\langle C_{n\downarrow} \rangle \equiv \frac{1}{V} \sum_{\vec{x}} \int \int 1 - \varphi_{\text{normalized}} dP_2 dS_c, \tag{54}$$

where $\overrightarrow{x}$ represents all discrete dimensions being summed over and V represents the size of the n-dimensional region under $\phi_{\text{normalized}}$. The coefficient $\langle C_{n\downarrow} \rangle$ can be used to visualize the average contribution of any controller modality and succinctly compactify a large amount of data into a single number.

The simulation shown in Figure 3g-h, 5h-j are based on this method.

I Base parameters and parameter rescaling

The following parameter values were used unless otherwise specified: $k_{m_{1,2}} = 3$, $k_{p_{1,2}} = 150$, $d_{m_{1,2}} = 1$, $d_{p_{1,2}} = 1$, $J_{m_{1,2}} = 1$, $J_{p_{1,2}} = 0.3$, $R_1 = 1$, $R_2 = 0\ 256$, $J_{1,2} = 0\ 100$, and $\Omega = 4$. $J_{p_{1,2}} = 0.2$ was used in Figure 1-2 and Supplementary Figure 1-3 for demonstration of the resource competitive noise (η_{RC}), while $J_{p_{1,2}} = 0.5$ was used in Figure 3-5 and Supplementary Figure 4-9 for demonstration of noise control. The maximum value of S_c was set to $S_c = 50$ when the noise reduction reaches saturation. The maximum value of R_2 was set to $R_2 = 256$ when the RFP mean reaches its saturation.

Parameter rescaling is done to ensure that the means of the mRNAs and proteins are the same across all cases, which is necessary for fair comparison of the noise behaviors of in all the models. All rescaling is done by resetting the transcription and translation rate constants (k_{m_i} and k_{p_i}) using the resource competition (RC) case as the base case in all figures except for Figure 2e-f, 5c, Sup Figure 3a-b, 7d-f, and 8c, where the orthogonal resources (OR) case was used as a base. The specific values of these rate constants for different systems are listed, as follows.

1. Unlimited Resources (UR) system:

$$k_{m_i} = \frac{d_{m_i} \langle m_i \rangle}{R_i}, \tag{55}$$

$$k_{p_i} = \frac{d_{p_i} \langle p_i \rangle}{m_i}. \tag{56}$$

2. Resource Competition (RC) system:

$$k_{m_i} = \frac{d_{m_i} \langle m_i \rangle \left(1 + \sum_j \frac{R_j}{J_{m_j}}\right)}{R_i}, \tag{57}$$

$$k_{p_i} = \frac{d_{p_i} \langle p_i \rangle \left(1 + \sum_j \frac{\langle m_j \rangle}{J_{p_j}}\right)}{\langle m_i \rangle}. \tag{58}$$

3. Orthogonal Resource (OR) system:

$$k_{m_i} = \frac{d_{m_i} \langle m_i \rangle \left(1 + \frac{R_i}{J_{m_i}}\right)}{R_i}, \tag{59}$$

$$k_{p_i} = \frac{d_{p_i} \langle p_i \rangle \left(1 + \frac{\langle m_i \rangle}{J_{p_i}}\right)}{\langle m_i \rangle}. \tag{60}$$

4. RC system with a controller:

$$k_{m_i} = \frac{d_{m_i} \langle m_i \rangle \left(1 + \sum_j \frac{R_j}{J_{m_j} C_{m_i}}\right)}{\frac{R_i}{C_{m_i}}}, \tag{61}$$

$$k_{p_i} = \frac{d_{p_i} \langle p_i \rangle \left(1 + \sum_j \frac{\langle m_j \rangle}{J_{p_j} C_{p_j}}\right)}{\frac{\langle m_i \rangle}{C_{p_i}}}, \tag{62}$$

where C_{m_i} and C_{p_i} are defined in Eqs. (44) and (45), respectively.

5. OR system with a controller:

$$k_{m_i} = \frac{d_{m_i} \langle m_i \rangle \left(1 + \frac{R_i}{J_{m_i} C_{m_i}}\right)}{\frac{R_i}{C_{m_i}}}, \tag{63}$$

$$k_{p_i} = \frac{d_{p_i} \langle p_i \rangle \left(1 + \frac{\langle m_i \rangle}{J_{p_i} C_{p_i}}\right)}{\frac{\langle m_i \rangle}{C_{p_i}}}. \tag{64}$$

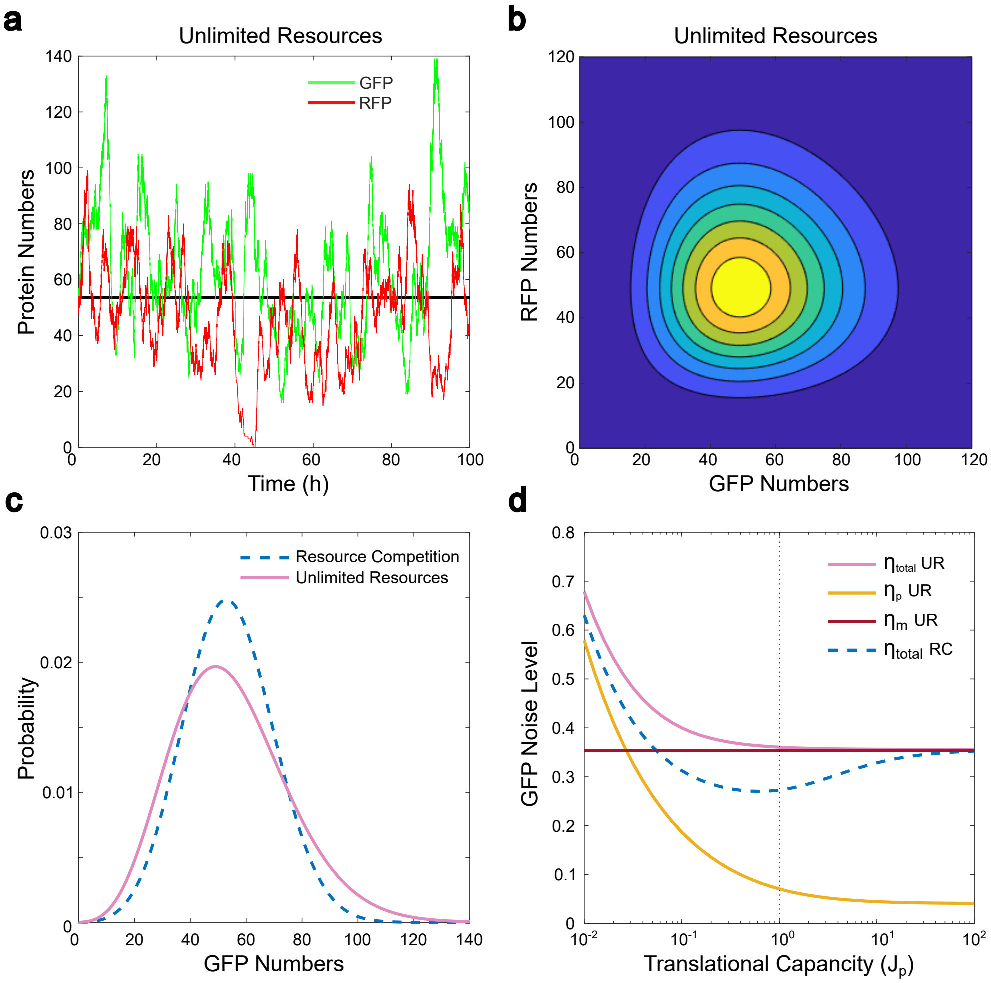

Supplementary Fig. 1: Reduction of gene expression noise by resource competition in comparison with the case of unlimited resources (UR). (a) Gillespie stochastic trajectories of GFP (green trace) and RFP (red trace) expression in a two-gene circuit with unlimited resources (correlation coefficient = -0.05). (b) The corresponding distribution of GFP and RFP expression levels from the solutions of the master equation. (c) Distribution of GFP expression levels in the systems with resource competition (blue dashed curve) and unlimited resources (pink curve). (d) The dependence of the GFP total noise level and its decomposition on the translational capacity J_p of limited resources in the host cell for the synthetic gene circuit. The GFP total noise level in the case with resource competition is shown in the dashed curve for comparison. Vertical line represents GFP noise levels for J_p value used in previous panels. The transcription and translation rate constants in the UR model are rescaled to ensure the same means of the mRNAs and proteins in the two models.

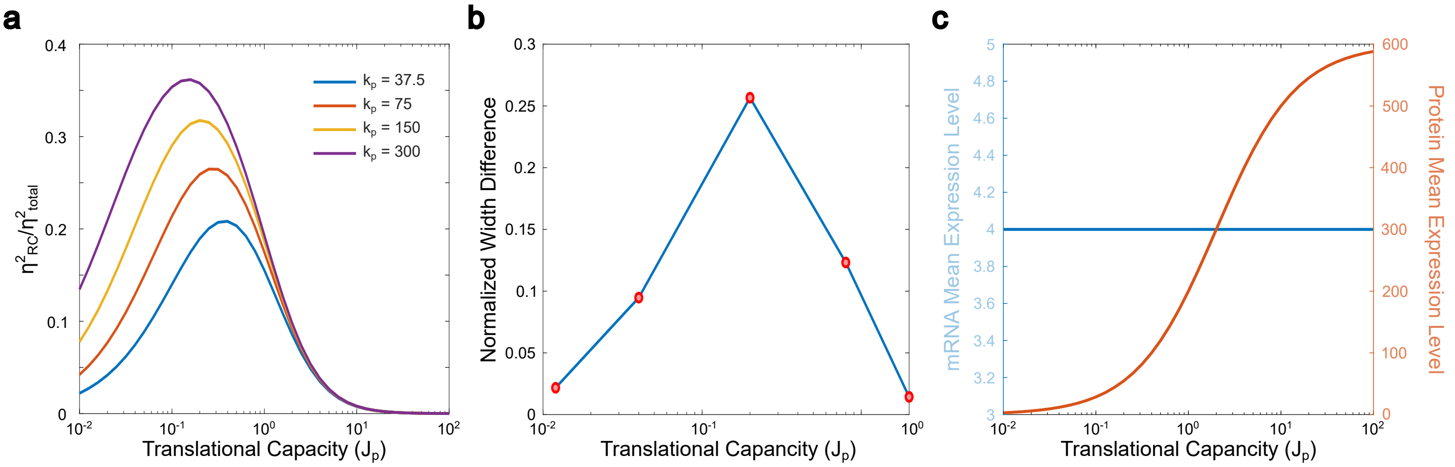

Supplementary Fig. 2: Effects of translational capacity on resource competition. (a) Percentage of RC noise at different protein production rates (k_p) as translational capacity (J_p) increases. (b) Percentage of changes in the width at half-heigh of probability distributions from stochastic Gillespie simulations for the system with and without RC noise as translational capacity (J_p) increases. (c) The dependence of mRNA and protein means on the translational capacity (J_p) for the RC system.

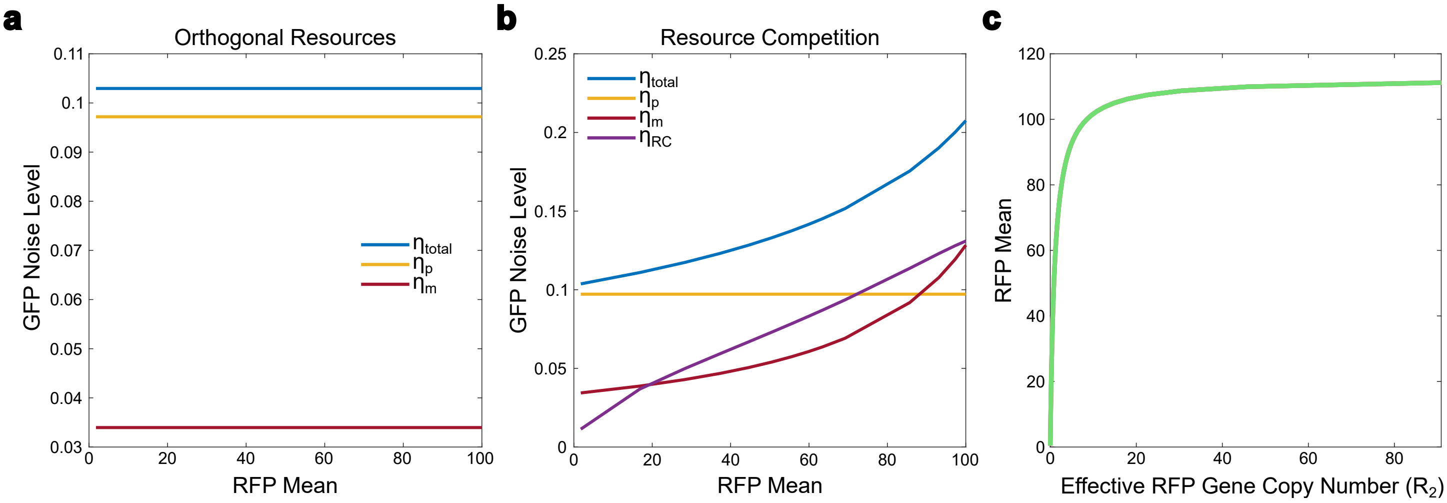

Supplementary Fig. 3: Analysis of protein noise decomposition in the system with and without orthogonal resources. (a) The dependence of the GFP total noise level and its two decompositions over RFP mean in the OR system. (b) The dependence of the GFP total noise level and its three decompositions over RFP mean in the RC system. (c) The dependence of the RFP means on effective rfp copy number.

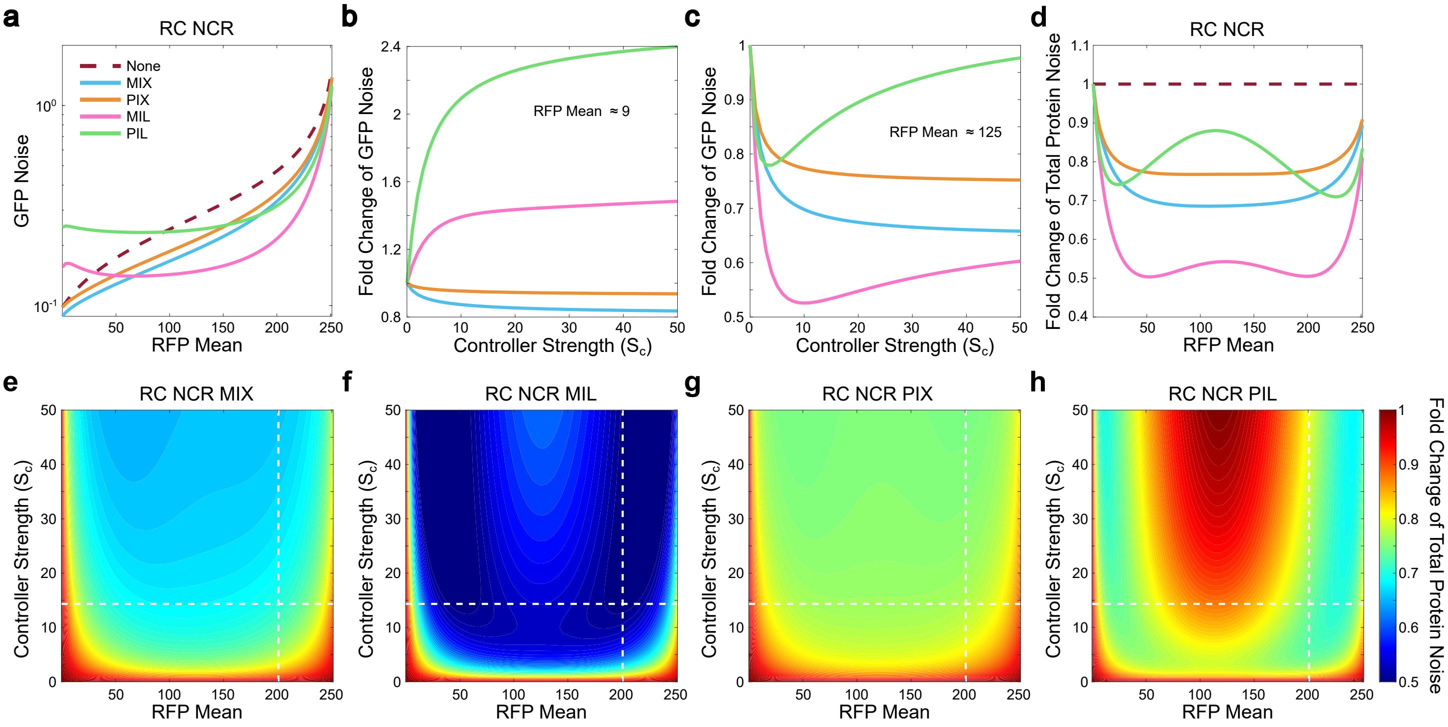

Supplementary Fig. 4: Analysis of the noise reduction by NCR controllers. (a) Dependence of the non-normalized GFP noise levels on RFP mean for an RC system with no controller (maroon dashed curve), an NCR-MIX controller (light blue curve), an NCR-PIX controller (orange curve), an NCR-MIL controller (pink curve), and an NCR-PIL controller (light green curve). (b, c) Dependence of normalized GFP noise on controller strength with respect to the RC system with no controller with a fixed RFP mean at 9 and 125, respectively. (d) FDT analysis demonstrating the normalized total protein noise (Pythagorean sum of GFP and RFP noise) on the RFP mean with NCR controllers applied in RC system for fixed controller strength $S_c = 14$. The noise levels are normalized to the base case without a controller. (e-h) Normalized total protein noise in the phase plane of RFP mean and NCR controller strength for NCR controller subtype (e) MIX, (f) PIX, (g) MIL, and (h) PIL. Deep blue color represents a strong decrease in the noise levels with respect to base case with no controller, and deep red indicates the absence of a significant noise change or even noise increase over the base case. Horizontal and vertical white dashed lines represent the controller strength and RFP mean used in panel a-d.

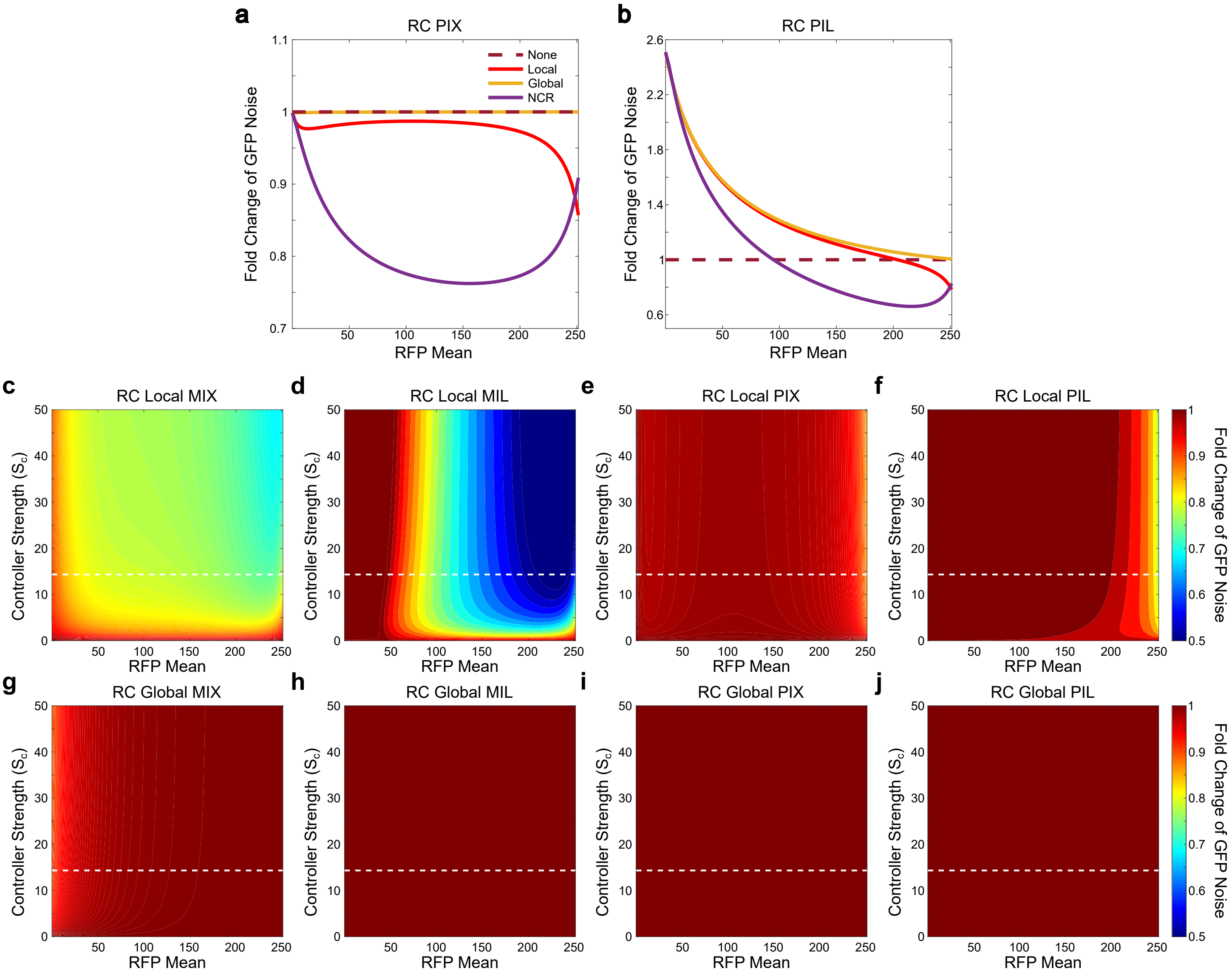

Supplementary Fig. 5: Analysis of noise reduction capability of global and local controllers. (a, b) Dependence of the normalized GFP noise levels on RFP mean for an RC system with PIX and PIL subtypes of three controllers, respectively. (c-j) Normalized GFP noise levels in the phase plane of RFP mean and controller strength for four subtypes of local controllers (c-f) or global controller (g-j). Deep blue region represents a strong decrease in noise levels in comparison to a resource competition case with no controller, and a deep red region represents a noise level increase or the absence of a noise change. The horizontal white dashed lines represent the controller strength in panel a-b.

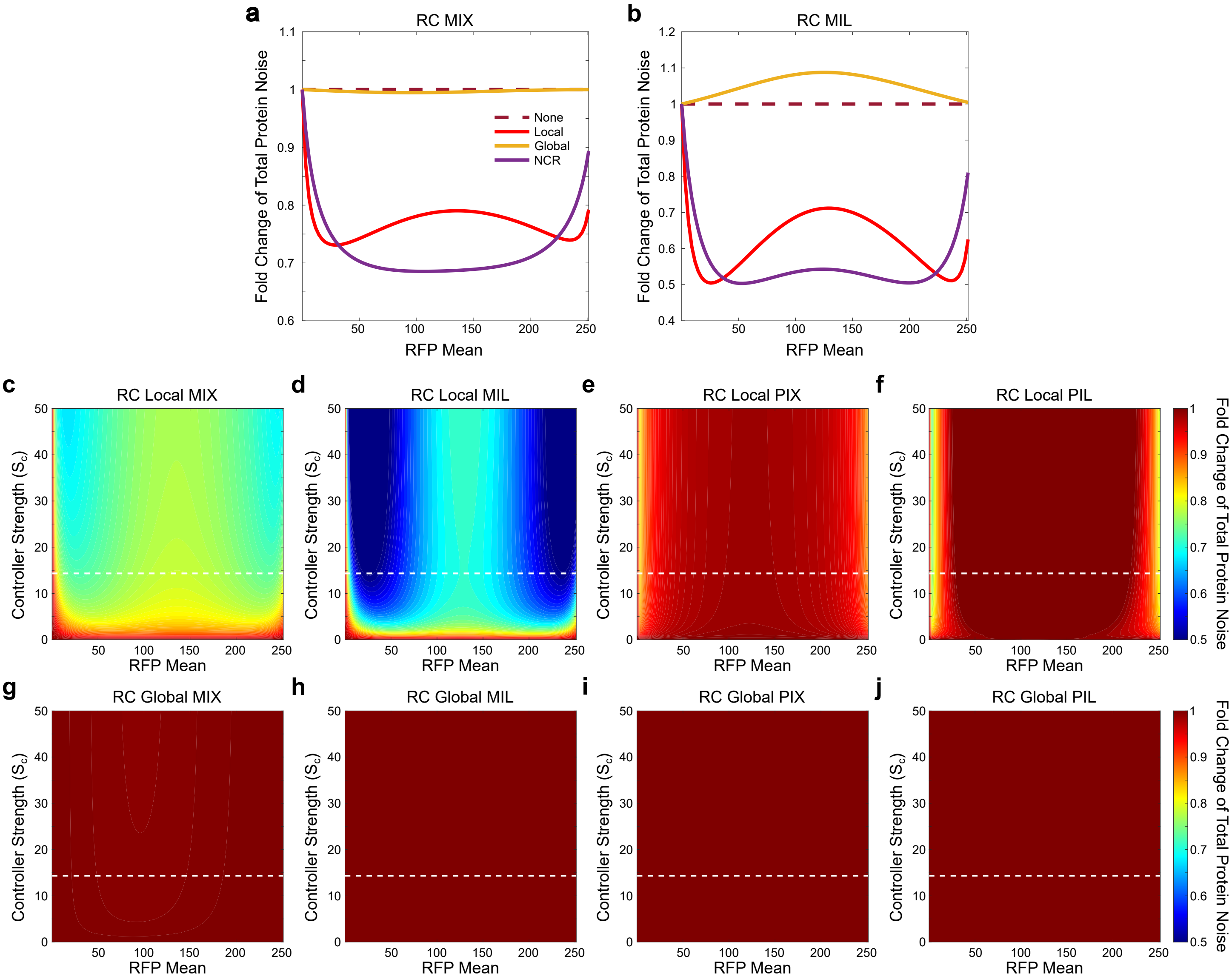

Supplementary Fig. 6: Analysis of the total protein noise reduction capability of local, global, and NCR controllers. (a,b) Dependence of the normalized total protein noise levels with local controllers (red curves), global controllers (yellow curves), and NCR controller (purple curves) using MIX and MIL subtypes respectively. The noise levels are normalized to the base case with no controller. (c-j) Normalized total protein noise levels in the phase plane of RFP mean and controller strength for four subtypes of local controllers (c-f) or global controller (g-j). Deep blue region represents a strong decrease in noise levels in comparison to a resource competition case with no controller, and a deep red region represents a noise level increase or the absence of a noise change. The horizontal white dashed lines represent the controller strength Figure 4c, 4d, Supplementary Figure 5a, and 5b.

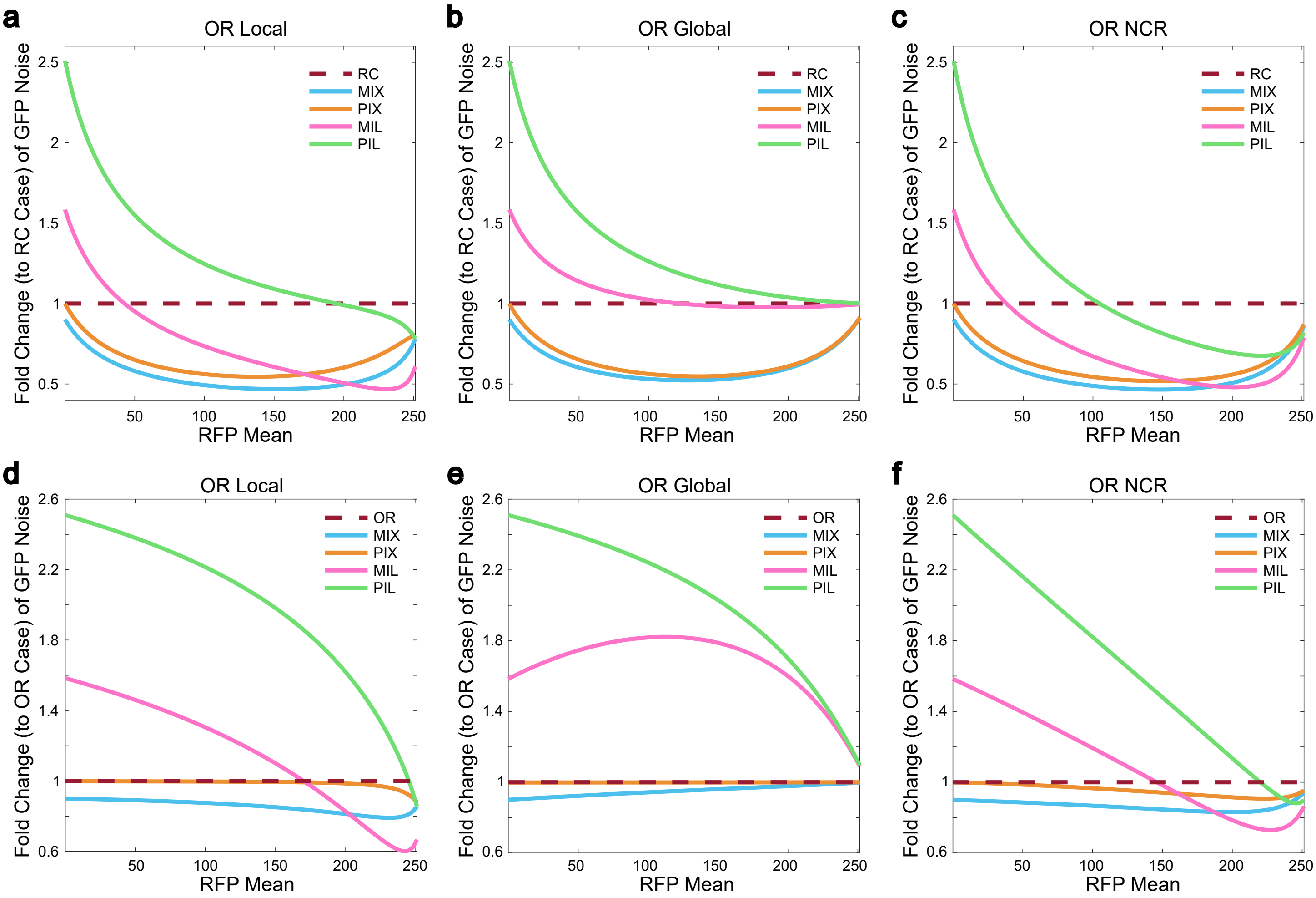

Supplementary Fig. 7: Analysis of the noise reduction by the negative feedback controllers combined with orthogonal resources. (a-c) Dependence of the normalized GFP noise levels on RFP mean for a system with four subtypes of a local controller, a global controller, and an NCR controller respectively. The GFP noise levels are normalized to the case with no controller and no orthogonal resources. (d-f) Same as a-b, but with the noise levels normalized to the case with orthogonal resources. The controller strength $S_c = 14$.

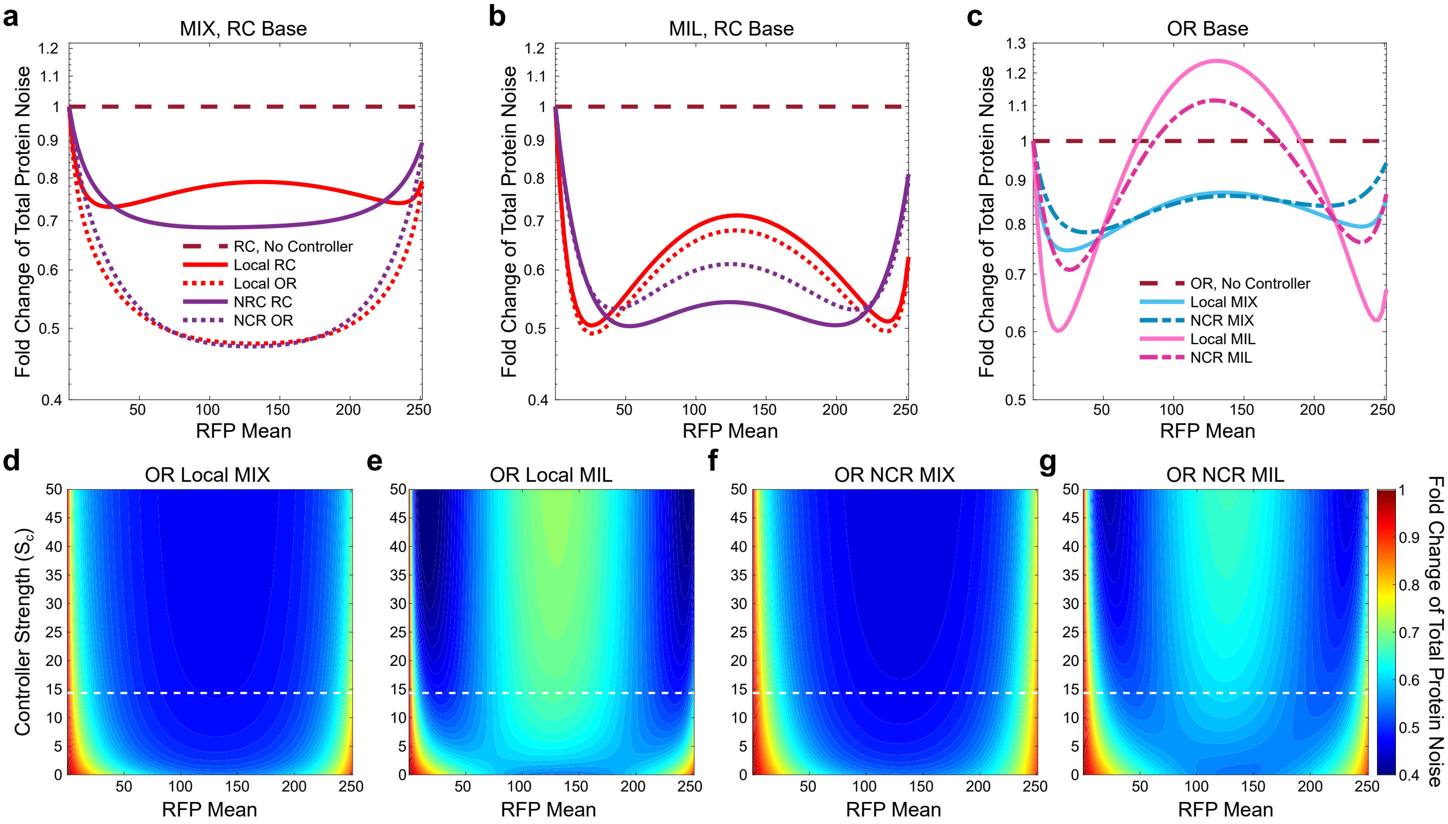

Supplementary Fig. 8: Control of total protein noise through combined negative feedback controllers and orthogonal resources. (a, b) Dependence of the normalized total protein noise levels on RFP mean with a local controller (red curves) and NCR controller (purple curves) combined with orthogonal resources (solid curves), respectively, for MIX and MIL controller subtypes. The dashed curves are cases without orthogonal resources or controllers. All data are normalized to those of the system without a controller (maroon dashed curve). (c) Dependence of the normalized total protein noise levels on RFP mean for the OR system combined with a local controller (solid curves) or an NCR controller (dashed curves) for either the MIX (blue curves) or MIL (fuchsia curves) controller subtypes. All data are normalized to those in the case with orthogonal resources but no controller (maroon dashed curve). (d-g) Normalized total protein noise in the phase plane of RFP mean and controller strength, respectively, for local-MIX, local-MIL, NCR-MIX, and NCR-MIL combined with orthogonal resources. Deep blue color represents regionsof higher noise reduction. The horizontal white dashed lines represent the controller strength shown in panel a-b.

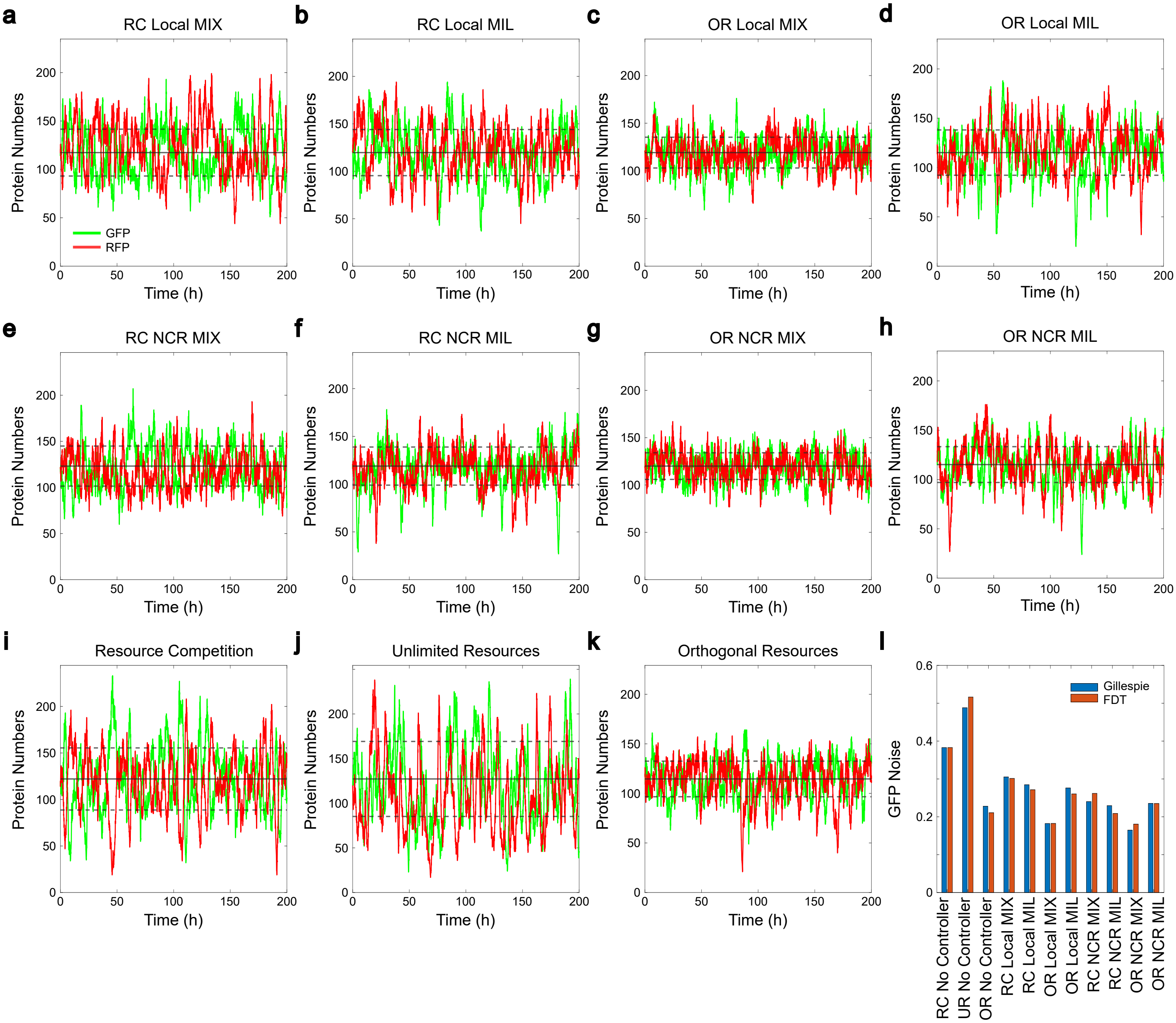

Supplementary Fig. 9: Gillespie stochastic trajectories of GFP (green trace) and RFP (red trace) expression for (a) RC local MIX case (correlation coefficient ≈ -0.77), (b) RC local MIL case (correlation coefficient ≈ -0.27), (c) OR local MIX case (correlation coefficient ≈ 0.1), (d) OR local MIL case (correlation coefficient ≈ 0.1), (e) RC NCR MIX case (correlation coefficient ≈ -0.59), (f) RC NCR MIL case (correlation coefficient ≈ 0.32), (g) OR NCR MIX case (correlation coefficient ≈ 0.14), (h) OR NCR MIL case (correlation coefficient ≈ 0.47), (i) resource competition (RC) case with no controllers (correlation coefficient ≈ -0.83), (j) unlimited resources (UR) case with no controllers (correlation coefficient ≈ 0.05), (k) orthogonal resources (OR) case with no controllers (correlation coefficient ≈ -0.22), and (l) GFP protein noise for each Gillespie stochastic simulation case versus that calculated from FDT analysis.

References

[1] A. Boo, T. Ellis, G. B. Stan, *Cur. Opin. Sys. Biol.* **2019**, *14* 66.

[2] O. Borkowski, F. Ceroni, G. B. Stan, T. Ellis, *Cur. Opin. Microbiol.* **2016**, *33* 123.

[3] D. D. Vecchio, *Tre. Biotech.* **2015**, *33* 111.

[4] A. Gyorgy, J. I. Jiménez, J. Yazbek, H. H. Huang, H. Chung, R. Weiss, D. D. Vecchio, *Biophys. J.* **2015**, *109* 639.

[5] Y. Qian, H. H. Huang, J. I. Jiménez, D. D. Vecchio, *ACS Syn. Biol.* **2017**, *6* 1263.

[6] R. Zhang, H. Goetz, J. Melendez-Alvarez, J. Li, T. Ding, X. Wang, X.-J. Tian, *Nat. Commun.* **2021**, *12* 853.

[7] M. B. Elowitz, S. Leibler, *Nature* **2000**, *403*, 6767 335.

[8] C. Zhang, R. Tsoi, L. You, *Integr. Biol.* **2016**, *8*, 4 456.

[9] N. A. Aleksashin, T. Szal, A. E. d'Aquino, M. C. Jewett, N. Vázquez-Laslop, A. S. Mankin, *Nat. Commun.* **2020**, *11* 1.

[10] W. An, J. W. Chin, *Proc. Natl. Acad. Sci. U.S.A.* **2009**, *106* 8477.

[11] A. P. S. Darlington, J. Kim, J. I. Jiménez, D. G. Bates, *ACS Syn. Biol.* **2018**, *7* 2485.

[12] A. P. S. Darlington, J. Kim, J. I. Jiménez, D. G. Bates, *Nat. Commun.* **2018**, *9* 695.

[13] D. L. Shis, M. R. Bennett, *Proc. Natl. Acad. Sci. U.S.A.* **2013**, *110* 5028.

[14] T. Frei, F. Cella, F. Tedeschi, J. Gutiérrez, G. B. Stan, M. Khammash, V. Siciliano, *Nat. Commun.* **2020**, *11* 1.

[15] T. W. Grunberg, D. D. Vecchio, *Cur. Opin. Biotech.* **2020**, *63* 41.

[16] A. Hamadeh, D. D. Vecchio, *53rd IEEE Conference on Decision and Control*, **2014** 3829–3834.

[17] H. H. Huang, Y. Qian, D. D. Vecchio, *Nat. Commun.* **2018**, *9* 1.

[18] G. Lillacci, Y. Benenson, M. Khammash, *Nuc. Acids Res.* **2018**, *46* 9855.

[19] T. Shopera, L. He, T. Oyetunde, Y. J. Tang, T. S. Moon, *ACS Syn. Biol.* **2017**, *6* 1596.

[20] A. Stone, R. Zhang, X.-J. Tian, *2021 American Control Conference (ACC)* **2021**,1882.

[21] F. Ceroni, A. Boo, S. Furini, T. E. Gorochowski, O. Borkowski, Y. N. Ladak, A. R. Awan, C. Gilbert, G. B. Stan, T. Ellis, *Nat. Methods* **2018**, *15* 387.

[22] M. B. Elowitz, A. J. Levine, E. D. Siggia, P. S. Swain, *Science* **2002**, *297* 1183.

[23] A. Hilfinger, J. Paulsson, *Proc. Nat. Acad. Sci. (USA)* **2011**, *108* 12167.

[24] M. Kaern, T. C. Elston, W. J. Blake, J. J. Collins, *Nat. Rev. Gene.* **2005**, *6* 451.

[25] B. Munsky, G. Neuert, A. V. Oudenaarden, *Science* **2012**, *336* 183.

[26] E. M. Ozbudak, M. Thattai, I. Kurtser, A. D. Grossman, A. V. Oudenaarden, *Nat. Gene.* **2002**, *31* 69.

[27] A. Sanchez, I. Golding, *Science* **2013**, *342* 1188.

[28] V. Shahrezaei, P. S. Swain, *Proc. Natl. Acad. Sci. U.S.A.* **2008**, *105* 17256.

[29] L. H. So, A. Ghosh, C. Zong, L. A. Sepúlveda, R. Segev, I. Golding, *Nat. Gene.* **2011**, *43* 554.

[30] M. Thattai, A. V. Oudenaarden, *Proc. Natl. Acad. Sci. U.S.A.* **2001**, *98* 8614.

[31] D. T. Gillespie, *Ann. Rev. Phys. Chem.* **2007**, *58* 35.

[32] J. Paulsson, *Nature* **2004**, *427* 415.

[33] J. Paulsson, *Phys. Life Rev.* **2005**, *2* 157.

[34] A. Bar-Even, J. Paulsson, N. Maheshri, M. Carmi, E. O'Shea, Y. Pilpel, N. Barkai, *Nat. Gene.* **2006**, *38* 636.

[35] J. R. S. Newman, S. Ghaemmaghami, J. Ihmels, D. K. Breslow, M. Noble, J. L. DeRisi, J. S. Weissman, *Nature* **2006**, *441* 840.

[36] Y. Taniguchi, P. J. Choi, G.-W. Li, H. Chen, M. Babu, J. Hearn, A. Emili, X. S. Xie, *Science* **2010**, *329* 533.

[37] S. Yang, S. Kim, Y. R. Lim, C. Kim, H. J. An, J.-H. Kim, J. Sung, N. K. Lee, *Nat. Commun.* **2014**, *5* 4761.

[38] A. Becskel, L. Serrano, *Nature* **2000**, *405* 590.

[39] M. Chen, L. Wang, C. C. Liu, Q. Nie, *ACS Syn. Biol.* **2013**, *2* 587.

[40] Y. Dublanche, K. Michalodimitrakis, N. Kömmerer, M. Foglierini, L. Serrano, *Mole. Sys. Biol.* **2006**, *2*.

[41] M. T. Guinn, G. Balázsi, *Nuc. Acids Res.* **2019**, *47* 7703.

[42] Z. Hensel, H. Feng, B. Han, C. Hatem, J. Wang, J. Xiao, *Nat. Str. Mole. Biol.* **2012**, *19* 797.

[43] A. Milias-Argeitis, M. Rullan, S. K. Aoki, P. Buchmann, M. Khammash, *Nat. Commun.* **2016**, *7* 12546.

[44] K. F. Murphy, R. M. Adams, X. Wang, G. Balázsi, J. J. Collins, *Nuc. Acids Res.* **2010**, *38* 2712.

[45] D. Nevozhay, R. M. Adams, K. F. Murphy, K. Josić, G. Balázsi, *Proc. Natl. Acad. Sci. U.S.A.* **2009**, *106* 5123.

[46] A. Singh, *IEEE Trans. Nanobiosci.* **2011**, *10* 194.

[47] L. Wang, J. Xin, Q. Nie, *PLOS Comp. Biol.* **2010**, *6* e1000764.

[48] I. Lestas, G. Vinnicombe, J. Paulsson, *Nature* **2010**, *467*, 174.

[49] J. Yan, A. Hilfinger, G. Vinnicombe, J. Paulsson, et al., *Phys. Rev. Lett.* **2019**, *123*, 108101.

[50] A. Martella, M. Firth, B. J. M. Taylor, A. Göppert, E. M. Cuomo, R. G. Roth, A. J. Dickson, D. I. Fisher, *ACS Syn. Biol.* **2019**, *8* 1998.

[51] A. Jolma, J. Zhang, E. Mondragón, E. Morgunova, T. Kivioja, K. U. Laverty, Y. Yin, F. Zhu, G. Bourenkov, Q. Morris, T. R. Hughes, L. J. Maher, J. Taipale, *Genome Res.* **2020**, *30* 962.

[52] S. Zhang, C. A. Voigt, *Nuc. Acids Res.* **2018**, *46* 11115.

[53] A. A. Smargon, Y. J. Shi, G. W. Yeo, *Nat. Cell Biol.* **2020**, *22* 143.

[54] D. B. T. Cox, J. S. Gootenberg, O. O. Abudayyeh, B. Franklin, M. J. Kellner, J. Joung, F. Zhang, *Science* **2017**, *358* 1019.

[55] M. P. Terns, *Mole. Cell* **2018**, *72* 404.

[56] C. J. Bashor, N. Patel, S. Choubey, A. Beyzavi, J. Kondev, J. J. Collins, A. S. Khalil, *Science* **2019**, *364* 593.

[57] D. J. McColl, C. D. Honchell, A. D. Frankel, *Proc. Natl. Acad. Sci. U.S.A.* **1999**, *96* 9521.

[58] M. Stamatakis, R. M. Adams, G. Balázsi, *Chaos* **2011**, *21* 047523.

[59] J. M. Raser, E. K. O'Shea, *Science* **2005**, *309* 2010.

[60] S. Klumpp, T. Hwa, *Cur. Opin. Biotech.* **2014**, *28* 96.

[61] S. Klumpp, Z. Zhang, T. Hwa, *Cell* **2009**, *139* 1366.

[62] Q. Liu, J. Schumacher, X. Wan, C. Lou, B. Wang, *ACS Syn. Biol.* **2018**, *7* 553.

[63] J. Melendez-Alvarez, C. He, R. Zhang, Y. Kuang, X.-J. Tian, *ACS Syn. Biol.* **2021**, *10* 1227.

[64] C. Tan, P. Marguet, L. You, *Nat. Chem. Biol.* **2009**, *5* 842.

[65] R. Zhang, J. Li, J. Melendez-Alvarez, X. Chen, P. Sochor, H. Goetz, Q. Zhang, T. Ding, X. Wang, X. J. Tian, *Nat. Chem. Biol.* **2020**, *16* 695.

[66] J. Lin, A. Amir, *Nat. Commun.* **2018**, *9* 4496.

[67] Y. Tanouchi, A. Pai, H. Park, S. Huang, R. Stamatov, N. E. Buchler, L. You, *Nature* **2015**, *523* 357.

[68] P. Thomas, G. Terradot, V. Danos, A. Y. Wei, *Nat. Commun.* **2018**, *9* 4528.

[69] R. D. Jones, Y. Qian, V. Siciliano, B. DiAndreth, J. Huh, R. Weiss, D. D. Vecchio, *Nat. Commun.* **2020**, *11* 1.

[70] C. L. Kelly, A. W. Harris, H. Steel, E. J. Hancock, J. T. Heap, A. Papachristodoulou, *Nucleic Acids Res.* **2018**, *46* 9875.

[71] C. Briat, A. Gupta, M. Khammash, *J. R. Soc. Interface* **2018**, *15*, 143 20180079.

[72] T.-L. To, N. Maheshri, *Science* **2010**, *327* 1142.

[73] A. Eldar, M. B. Elowitz, *Nature* **2010**, *467* 167.

[74] M. Thattai, A. Van Oudenaarden, *Genetics* **2004**, *167* 523.

[75] E. Kussell, S. Leibler, *Science* **2005**, *309* 2075.

[76] M. Acar, J. T. Mettetal, A. Van Oudenaarden, *Nat. Genet.* **2008**, *40* 471.

[77] W. J. Blake, G. Balázsi, M. A. Kohanski, F. J. Isaacs, K. F. Murphy, Y. Kuang, C. R. Cantor, D. R. Walt, J. J. Collins, *Mol. Cell* **2006**, *24* 853.
